# Cross verification of prescribing trends through loop evaluation of physicians, patients and medical store personnel

**DOI:** 10.1186/s12913-019-4145-7

**Published:** 2019-05-22

**Authors:** Syed Zia Husnain, Nadeem Irfan Bukhari, Khalid Hussain, Zaheer-Ud-Din Babar, Furqan Khurshid Hashmi, Zikria Saleem, Muhammad Salman, Louise Curley

**Affiliations:** 1 Punjab University College of Pharmacy, University of the Punjab, Allama Iqbal Campus, Lahore, 54000 Pakistan; 2 Department of Pharmacy, University of Huddersfield, Queensgate, Huddersfield, UK; 3 School of Pharmacy, Faculty of medical and health sciences, University of Auckland, Auckland, New Zealand

**Keywords:** Prescribing trends, Prescribing guidelines, Generic prescribing, Rational prescribing practices, Multinational branded medicines

## Abstract

**Background:**

Prescription connects physician, patient and community pharmacy personnel who can help in understanding prescribing pattern. The present study was aimed to get an insight of viewpoints of all members involved in progression of events from prescription to drug purchase, i.e., physician, patient and medical-store personals regarding the prescription pattern in Pakistan.

**Methods:**

Therefore, a cross-sectional study was conducted in four provinces and capital territory (Islamabad) of Pakistan to evaluate the perception of physicians, patients and medical stores/pharmacy personnel of the prescribing trends in Pakistan.

**Results:**

Response rate was higher from Punjab and lower in Sindh. Responses of 981 walk-in patients with 393 physicians and 618 medical stores/pharmacies were received and statistically evaluated. The majority of physicians, patients and pharmacists/medical store personnel considered the medicines of multinational manufacturers as more effective. Physicians considered their prescribing cost-effective. However, majority of patients as well as pharmacists/medical store personnel strongly disagreed or disagreed with this notion that physicians prescribe cheap medicines. Furthermore, physicians and patients reported that medicines of local companies were not as effective as the medicines of multinational manufacturers, which were contrary to what pharmacists thought. Majority of physicians disagreed that their prescribing was under the influence of medical stores in their vicinity. The response of most of the patients (40.5%) was in line with that of physicians whereas 32% pharmacist/medical store personnel agreed. Nearly half of the physicians strongly agreed or agreed that patients demand medicines of multinational companies. Contrarily, a majority of patients and medical store personnel denied that patients demand for the medicines of multinational manufacturers.

**Conclusion:**

The study highlighted that there was a need to develop policy guidelines at the level of Federal Government and Drug Regulatory Authority of Pakistan in connection with prescribing practices to reduce the variation in perception of key stakholders involved in drug use process.

## Background

A prescription is the form of instructions, written by the physician that governs the care plan for a patient to be performed either by a patient himself or his caretaker, nurse, pharmacist or another therapist [1]. Prescribing is an essential part of physician’s daily routine work and is a professional and ethical responsibility [2]. The most appropriate drugs for patients should be selected according to the guidelines laid down by the World Health Organization (WHO) [3] that includes the comparative effectiveness, safety, convenience and cost [4–6].

Prescription is a document which links doctor, patient and a community pharmacy (medical store). Beyond that, a prescription may be a documented record that subsequently be evaluated for drug utilization for the clinical, educational and economic purposes [7]. Prescription writing requires professional skills and updated knowledge of the prescriber and reflects the clinical skills and behavior of the prescriber [8].

Appropriateness in the healthcare is the outcome of a process of decision making that maximizes net individual health gains within society’s available resources [9]. This concept implies that the patient’s attitude is an important deciding factor in the appropriateness of the prescribed medicines and success of healthcare delivery [10]. Prescribing is not an easy task and data reveal a range of poor prescribing (such as medication errors, under-prescribing, over prescribing, inappropriate or irrational prescribing) by physicians in different settings [11]. Lack of compliance to the essential drug list (EDL) and the standard treatment guidelines is a common trend in practice [12]. The issue of irrational prescribing and inappropriate use of drugs is pivotal and a large number of studies involve prescribing practices in the public sector, while the irrational use of drugs is also prevalent in private sector [12].

The magnitude of inappropriate drug use at the community level is often overlooked, and the perspective of consumers/patients is hardly addressed. It is imperative to include the patient perspective, including the socio-cultural factors influencing the impact of drug use behavior. The viewpoint of pharmacies/medical stores also needs exploration in factors influencing rational prescribing as well as appropriate use of drugs in order to ensure the good quality continuum of care to the consumers/patients.

Keeping the above in view, the current study was planned to take standpoint of all the members and items involved in the cascade of events, i.e., prescribers (physicians), prescription, patients of same physicians bearing prescription and the persons in command at the point of purchase of medicines by patients (the medical stores) in the close vicinity of the prescribers. Agreement (or otherwise) between all the three groups for similar quarries on prescription parameters was noted along with recording of the demographic data of the study population. This is the first study of its kind in Pakistan considering transfer and sequence of events from prescriber to medical store in a step-wise manner.

## Methods

### Study design and population

This cross-sectional study was conducted in four provinces (Punjab, Sindh, Baluchistan and Khyber Pakhtunkhwa) and capital territory (Islamabad) of Pakistan, during a period of 11 months (March, 2015-January, 2016). Registered medical practitioners (RMPs/physicians) from both public and private healthcare facilities, walk-in patients (Conscious, mentally fit patients of both genders with age > 18 years) of the same prescriber and licensed medical stores/pharmacies of nearby healthcare facilities were eligible for inclusion in the study. Non-adult patients and individuals who did not give the consent were excluded from the study. The information regarding the RMPs and medical stores (MS) of the whole country was obtained from the Pakistan Medical and Dental Council (PMDC) and the Pakistan Pharmacy Council (PPC), respectively.

### Sample size

Based on the size of the population of physicians, patients and the medical store in the country, the sample size was considered from a table already published in NEA Research Bulletin [18], according to which the sample size for a population of 1,000,000 or above would be 384. However, keeping in view the possible reduction in response, we distributed a higher number of questionnaires than the required sample size.

### Outcome measures

Three separate sets of questionnaires were developed for the physician, patients and medical store personnel, using information from the literature review [13–17]. The study instruments were composed of 2 sections; Section A to gather demographic data of doctors, MS/pharmacists and patients, Section B had 8-items to assess the perception of study participants regarding prescription, prescribed drugs and prescribing trends. A 5-point Likert Scale (strongly agree to strongly disagree) was used for all items assessing the perception about prescription, prescribed drugs and prescribing trends. Responses were ranked in order of strength, i.e., strongly agree = 5, agree = 4, neither agree nor disagree (being neutral) = 3, disagree = 2 and strongly disagree = 1.

Face and content validity were performed by two experts (NIB and FKH) from the Punjab University College of Pharmacy, University of the Punjab, Lahore, Pakistan, who were experienced in conducting quantitative studies, especially in the areas of medication use, pharmacy practice and pharmaceutical policy. The questionnaire was revised according to the suggestions and comments received. The questionnaires focusing on MS-personnel and patients were translated into Urdu, national language of Pakistan, by forward and back translation from two English and Urdu language experts, respectively. After translation, the instruments were pilot tested for clarity and understanding among 5 patients and 5 MS personnel. All participants stated that the items on the study instrument were clear and understandable.

### Data collection

A convenient sampling method was used and trained researchers approached and explained the study objectives to pharmacists/MS personnel and physicians. Moreover, researchers also approached the patients after acquiring permission from the respective physicians. Those consented to participate in the study were administered the questionnaire. Data collection pattern for the current study was 1 Doctor (from every area visited by the researcher), 3 of his/her Patients and 2 pharmacists or MS personnel in the close vicinity of doctor’s clinic/hospital.

### Statistical analysis

The data were entered into the Statistical Package for the Social Sciences® (SPSS) version 22.0 for Windows. Percentages and frequencies were used for categorical variables, while median and range were calculated for the continuous variables. K-S tests were performed, which showed the non-normal distribution of data. Therefore, we performed Kruskal-Wallis H Test as well as *post-hoc* analysis to compare the responses of study participants regarding queries to assess their perception about prescription, prescribed drugs and prescribing trends.

## Results

As shown in Table 1, a total of 393 physicians, their 981 walk-in patients and 618 medical stores/pharmacies responded to the questionnaire distributed. The total response rates for the physician, patients and medical store persons were 81.4, 67.7 and 63.97%, respectively.

**Table 1:** Response rate of the participants from Punjab, Sindh, Khyber Pakhtunkhwa, Baluchistan and Islamabad capital territory

Province/area	Population	Questionnaire distributed	Number responded	Percent response (%)
Punjab	Physician	250	224	89.6
	Patient	750	579	77.2
	Medical Store	500	378	75.6
Sindh	Physician	125	77	61.6
	Patient	375	175	46.7
	Medical Store	250	105	42.0
Khyber Pakhtunkhwa	Physician	63	56	88.8
	Patient	189	132	69.8
	Medical Store	126	75	59.5
Baluchistan	Physician	30	23	76.6
	Patient	90	61	67.8
	Medical Store	60	39	65.0
Islamabad Capital Territory	Physician	15	13	86.6
	Patient	45	34	75.6
	Medical Store	30	21	70.0

Demographic data of physicians, patients and MS personnel are presented in Tables 2, 3 and 4, respectively. There was a predominance of male physicians having age < 30 years followed by 31–40 years from private hospitals. Moreover, there was a majority of 18–30 years old male patients belonging to the middle economic class in our sample. Regarding demographics of MS/pharmacy personnel, there was a preponderance of male pharmacists having 1–20 year experience and was filling 51–80 prescriptions per day.

**Table 2:** Demographics of the physicians (*n* = 393)

Parameter	N (%)
Gender	
Male	318 (80.9)
Female	75 (19.1)
**Age**	
18–30	116 (29.5)
31–40	107 (27.3)
41–50	70 (17.8)
51 and above	52 (13.2)
No answer	48 (12.2)
Specialty	
Dermatology	12 (3.1)
Family Physician	3 (0.8)
GP	36 (9.2)
Gynae	12 (3.1)
Pediatrics	4 (1.0)
Cardiology	8 (2.0)
Neurology	8 (2.0)
Dental	4 (1.0)
Medicine	64 (16.3)
Trainees	98 (24.9)
No answer	144 (36.7)
Education	
MBBS	237 (60.3)
FRCS	4 (1.0)
FCPS	121 (30.8)
House officer	18 (4.6)
No answer	13 (3.3)
Service type	
Government	149 (37.9)
Private	223 (56.7)
No answer	21 (5.4)
Experience	
1–10	26(6.6)
11–20	14(3.6)
21–30	64(16.3)
Above 30	25(6.4)
No answer	264(67.2)
Prescription written / day	
1–10	43 (10.9)
11–15	135 (34.4)
16–20	87 (22.1)
> 20	107 (27.2)
No answer	21 (5.4)
Working hours / day	
6	197 (50.1)
5	19 (4.8)
4	28 (7.1)
3	8 (2.0)
2	4 (1.0)
> 6	120 (30.5)
No answer	17 (4.4)
Consultation time /patient (minutes)	
1–5	116 (29.5)
6–10	46 (11.7)
11–15	68 (17.3)
16–20	64 (16.3)
> 20	9 (2.3)
No answer	90 (22.9)
Consultation fee (PKR)	
≤ 500	90 (22.9)
501–1000	55 (14.0)
> 1000	171 (43.5)
Public sector	34 (8.7)
Did not disclose	43 (11.0)

**Table 3:** Demographics of patients (*n* = 981)

Parameter	N (%)
Gender	
Male	614 (62.6)
Female	367 (37.4)
Age	
18–30	622 (63.4)
31–40	138 (14.1)
41–50	87 (8.8)
51 and above	131 (13.4)
No answer	3 (0.3)
Education	
Primary	225 (22.9)
Secondary	273 (27.8)
Higher secondary	259 (26.4)
Graduation	6 (0.6)
Other	191 (19.5)
Uneducated	27 (2.7)
Not replied	–
Locality	
Posh	72 (7.3)
Middle	639 (65.1)
Poor Mixed Not replied	204 (20.8) 54 (5.5) 12 (1.2)
Consultation fee (PKR)	
≤ 500	209 (21.3)
501–1000	237 (24.3)
> 1000	256 (26.1)
Public sector	171 (17.4)
No answer	108 (11.0)
Duration of Checkup (minutes)	
21–30	45 (4.6)
16–20	114 (11.6)
11–15	162 (16.5)
6–10	273 (27.8)
1–5	156 (15.9)
Other	213 (21.7)
No answer	18 (1.8)

**Table 4:** Demographics of medical store personnel (*n* = 618)

Parameter	N (%)
Gender	
Male	605 (97.7)
Female	13 (2.1)
Age	
18–30	225 (36.4)
31–40	132 (21.4)
41–50	129 (20.9)
51 and above	39 (6.3)
No answer	93 (15)
Education	
Secondary	186 (30.1)
Graduation	153 (24.8)
Pharmacy	261 (42.2)
No answer	18 (2.9)
Experience (years)	
1–10	132 (21.4)
11–20	221 (35.8)
21–30	177 (28.6)
Above 30	88 (14.2)
No answer	–
Locality	
Posh	69 (11.2)
Middle	438 (70.9)
Poor	66 (10.7)
Mixed	30 (4.9)
Not replied	15 (2.4)
Opening hours	
24	15 (2.4)
18	96 (15.5)
12	411 (66.5)
< 12	84 (13.6)
No answer	12 (1.9)
Prescription filled /day	
1–50	88 (14.3)
51–80	177 (28.6)
81–99	44 (7.1)
> 100	309 (50)

Responses of study participants to questions regarding the perception of prescription, prescribed drugs and prescribing trend are shown in Table 5. The majority of physicians, patients and pharmacists/MS personnel reported that the medicines of multinational manufacturers were more effective. On inclination of physicians to prescribe cost-effective medicines, the physician response was divided as 37.1% physicians strongly agreed or agreed whereas 32.3% were neutral and 8.4% did not reply. However, most of the patients as well as pharmacists/MS personnel strongly disagreed or disagreed that physicians prescribe cheap medicines. Majority of the physicians and patients reported that the medicines of multinational and local companies were not equally effective. By contrast, pharmacists agreed upon the equal effectiveness of multinational and local companies. Around 65% physicians, 57% patients and 56% MS personnel agreed or strongly agreed that physicians followed ideal prescription practices. Most of the physicians and pharmacists reported that high-price locally manufactured medicines were not effective. However, 38.7% patients strongly disagreed or denied the above. Around 64% of the physicians strongly disagreed or disagreed that they prescribed the medicines under influence of the MS personnel. The response of most of the patients (40.5%) was in line with that of the physicians while 32% MS personnel either strongly agreed or agreed. On the other hand, 22.4% physician, 21.8% patients and 50% MS personnel, either remained neutral or did not respond to this question. Nearly half of the physicians strongly agreed or agreed that patients demanded medicines of multinational companies. On the contrary, majority of patients and MS personnel denied that patients demand for medicines from the multinational manufacturers. Inter-group comparisons of responses to the questions assessing perception about prescription are shown in Table 6.

**Table 5:** Perception of doctors, patients, and medical store/pharmacy personnel about prescription, prescribed drugs and prescribing trends

Prescription aspects n (%)	Participants	SA	A	N	D	SD	NR
Medicines of multinational companies are more effective	**Physician**	127 (32.3)	209(53.2)	20(5.1)	24 (6.1)	4 (1.0)	9 (2.3)
	**Patient**	368 (37.5)	372(37.9)	174(17.7)	46 (4.7)	6 (0.6)	15 (1.5)
	**Medical store**	288 (46.6)	153(24.8)	63(10.2)	48 (7.8)	39 (6.3)	27(4.4)
Physicians are inclined to prescribe the economical medicines	**Physician**	52(13.2)	94(23.9)	127(32.3)	67 (17.0)	20 (5.1)	33 (8.4)
	**Patient**	84 (8.6)	160(16.3)	90(9.2)	322 (32.8)	279 (28.4)	46 (4.7)
	**Medical store**	147 (23.8)	87(14.1)	105(17.0)	144 (23.3)	120 (19.4)	15 (2.4)
Medicines of multinational and local companies are equally effective	**Physician**	24 (6.1)	64(16.3)	123(31.3)	107 (27.2)	54 (13.7)	21 (5.4)
	**Patient**	57 (5.8)	228(23.2)	237(24.2)	366 (37.3)	48(4.9)	45(4.6)
	**Medical store**	201 (32.5)	87(14.1)	96(15.5)	141 (22.8)	72(11.7)	21 (3.4)
Medicine of multinational companies are prescribed more due to their availability at nearby pharmacy	**Physician**	48(12.2)	159(40.5)	85(21.6)	48 (12.2)	12 (3.1)	41 (10.4)
	**Patient**	261 (26.6)	456(46.5)	72(7.3)	118 (12.0)	38(3.9)	36 (3.7)
	**Medical store**	213 (34.5)	198(32.0)	48(7.8)	75(12.1)	66 (10.7)	18 (2.9)
Rational prescription practices are being followed by physicians	**Physician**	105(26.7)	151(38.4)	100(25.4)	12 (3.1)	00 (0.00)	25 (6.4)
	**Patient**	174 (17.7)	384(39.1)	149(15.2)	178 (18.1)	66 (6.7)	30 (3.1)
	**Medical store**	219 (35.4)	129(20.9)	105(17.0)	93 (15.0)	60 (9.7)	12 (2.0)
High-prices local medicines are also as effective as multinational medicines	**Physician**	24 (6.1)	48(12.2)	116(29.5)	129 (32.8)	47 (12.0)	29 (7.4)
	**Patient**	60 (6.1)	320(32.6)	258(26.3)	245 (25.0)	66 (6.7)	32 (3.3)
	**Medical store**	123 (19.9)	87(14.1)	81(13.1)	198 (32)	105 (17)	24 (3.9)
A liaison exists between impact of physician to influence the dispensing pattern of local pharmacy	**Physician**	11 (2.8)	44(11.2)	47(12.0)	172 (43.8)	78 (19.8)	41(10.4)
	**Patient**	111 (11.3)	259(26.4)	178(18.1)	286 (29.2)	111(11.3)	36 (3.7)
	**Medical store**	75 (12.1)	123(19.9%)	114(18.4)	81 (13.1)	30 (4.9)	195(31.6)
Patient demand medicines of multinational companies	**Physician**	51(13.0)	140(35.6)	91(23.2)	62 (15.8)	28 (7.1)	21 (5.4)
	**Patient**	81 (8.3)	148(15.1)	130(13.3)	321 (32.7)	243 (24.8)	58 (5.9)
	**Medical store**	111 (18)	87(14.1)	63(10.2)	165 (26.7)	180(29.1)	12 (2.0)

**Table 6:** Inter-population deviation in responses and perception about prescription

Prescription aspects	Inter-group comparison		*p* value*
Medicines of multinational companies are more effective	Patients	Medical Store	0.600
		Physician	0.692
	Physician	Medical Store	0.543
Physicians are inclined to prescribe the economical medicines	Patients	Medical Store	< 0.001
		Physician	**<** 0.001
	Physician	Medical Store	0.105
Medicines of multinational and local companies are equally effective	Patients	Medical Store	< 0.001
		Physician	< 0.001
	Physician	Medical Store	< 0.045
Medicine of multinational companies are prescribed more due to their availability at nearby pharmacy	Patients	Medical Store	0.912
		Physician	< 0.001
	Physician	Medical Store	< 0.001
Rational prescription practices are being followed by physicians	Patients	Medical Store	< 0.001
		Physician	< 0.001
	Physician	Medical Store	0.179
High-prices local medicines are also as effective as multinational medicines	Patients	Medical Store	< 0.001
		Physician	< 0.001
	Physician	Medical Store	0.037
A liaison exists between impact of physician to influence the dispensing pattern of local pharmacy	Patients	Medical Store	< 0.001
		Physician	< 0.001
	Physician	Medical Store	0.019
Patient demand medicines of multinational companies	Patients	Medical Store	0.291
		Physician	< 0.001
	Physician	Medical Store	< 0.001

* Kruskal-Wallis H test

## Discussion

This study was sought to assess the perception of doctors, patients of the same prescriber and pharmacists/MS personnel regarding prescription, prescribed drugs and prescribing trend. It was a general perception of the majority of the physicians, MS personnel and patients that the medicines manufactured by the multinational companies were more effective (*P* > 0.05). The absence of system in Pakistan to regulate the prescription of generic medicines to the patients has led the patients to believe in general that multinational brands are more effective. Previous data indicated that physicians were important driving factor in determining whether patients receive either branded or generic drugs [19]. The pharmaceutical industry is one of the most important factors that affect physician’s prescribing decisions and prescribing behavior [20]. Physicians who believe in that they receive accurate information from pharmaceutical sales representatives show higher expenditures on branded drugs [21]. Our study suggests that there should be some standard guidelines in Pakistan for the prescription of medicines and in addition to that the generic prescribing system should be in place in the country. Similar findings were presented by Chua and co-worker in Malaysia, emphasizing the availability of national guidelines for the prescription of generics [22]. Patient’s trust is a key component of the patient-physician relationship. However, a study demonstrated that patient trust in the physician is not associated with the likelihood that a service is requested or provided during the visit, but prescription of a new medication was more frequent among regularly visiting patients to the same physician [23].

In the query regarding equal effectiveness of medicines of local and multinational companies, we found that MS persons believed that both categories of drugs were equally effective but physicians and the patients contradicted it. Opinions of physicians and patients could be attributed to the faith of the three stakeholders in the medicines of multinational origin. Different point of view of MS persons might be due to brand replacement practiced at medical store level. Sometimes MS persons, instead of losing their customer due to non-availability of a particular brand, replace the brand by their own considering prescribed and the replaced brands were equally effective. Regarding the perception about the prescribing of cheaper medicines, opinions of patients and MS persons were different from that of the physicians. Prescribing costly brands of medicines increase the economic burden on patients even after the expiration of patent [24–26]. In Pakistan, the prescription of expensive medicines would add to the economic burden on the patients where 80% of the health expenses are paid directly from the pocket of the consumers. Educating patients and their doctors enables them to reduce the cost of treatment by rationally selecting the medicines [27]. Andrea Coscelli found a significant evidence of time-dependence in prescription choices for physicians as well as patients, which seemed to imply that in molecular submarkets in which brands were not allowed to compete based on price, physician and patient ‘habit’ at the micro-level could translate into sticky and persistent market shares at the aggregate level [28].

According to the majority of physicians and MS personnel medicines of multinational companies are prescribed more frequently because these are more available at the nearby pharmacies. This implies that the multinational companies ensure the availability of their registered drugs as required under the Drugs Act 1976. The different response of physicians from that of patients and MS persons for the above aspect indicates that physicians contemplate the state of disease and prescribe drugs based on the diagnosis instead of considering just the availability. In contrast, the patients concerned about the availability of required drugs prescribed and MS persons also linked drug distribution to patients hence responses of patients and medical store personnel were similar in this study.

Prescribing is a complex task that requires diagnostic skills, knowledge of medicines, understanding of the clinical pharmacology principles, communication skills and ability to appreciate risks and uncertainties [29, 30]. The rational prescribing should attempt to maximize clinical effectiveness, minimize harm, avoid wasting scarce healthcare resources and respect patient choice [30]. Keeping the aspects of rational prescribing practices in view, the present study demonstrated variations among the participants of the study. Physicians viewed that they adhered to the rational prescription practices, which was not endorsed by the patients and MS persons. However, knowledge of patients about rational prescription is considered to be a limitation factor [26]. Prescribers predominantly considered that sometimes patients demand medicines from the multinational companies, but patients and MS persons refuted this notion of physicians. Since the prices of medicines from the multinational companies are higher in Pakistan than the local drugs hence the patients’ demand to prescribe these particular medicines, causes cost burden of high-price drugs on the shoulders of the patients. Federal Government has approved prices of drugs manufactured by multinational companies in Pakistan higher than the local drugs. Different perception of medical MS personnel regarding prices of drugs may be due to practice of brand switching for more profits.

The influence of physicians to impact the dispensing patterns in pharmacies in close vicinity to their clinics was also observed. The results suggested that physicians, patients and MS persons did not have the same perceptions because the patients and MS personnel perceived that dispensing patterns of medicines from pharmacies in nearby to physicians’ clinics were affected by the local general practitioners. Prescribing trends of physicians have been assessed in various studies, showing growing body of evidence explaining the determinants of physicians’ prescription choices between different versions of the same drug [31]. It is reported that the physicians are important agents in the choice of a drug [19]. Both physician’s habits and patient’s preferences are the most important factors that determine whether or not the later receive either brand name or generic drug [28]. It has also been reported that the existence of moral hazard in prescription behavior in the sense that physicians are more likely to prescribe branded drugs to their patients if patients get most of their costs reimbursed [32]. It is the incentive paid to the physicians that drives their prescribing attitude [33]. In Pakistan, due to lack of co-payment system and third-party payment system, the burden has to be borne by the patients for their medication in addition to adverse health outcomes due to irrational prescriptions.

A good body of evidence supports the aspect as the most important factor of generic substitution is the magnitude of saving money [34–36]. The experience of customers about generic medicines is reported to be positively associated with their willingness to accept generic substitution [37]. Patients with chronic disease conditions have lower acceptability rates of generic medicine substitution [38]. In Portugal, studies had concluded that the endorsement of generic drugs was significantly lower for illness labels which were perceived as more serious and that beliefs about efficacy were significantly affected by age and level of education [39, 40].

This study had few limitations. We only gathered the data from cities and towns of the four provinces and Islamabad capital territory of Pakistan. We did not collect the data from rural areas and also Gilgit Baltistan province of Pakistan so our sample may not be the representative of the entire population. Moreover, we did not use a non-probable sampling method such as randomization, therefore we had disadvantages such as selection biasness and non-generalizability.

In Australia, a very successful project namely, The Veterans Medicine Advice and Therapeutic Education Services (MATES) has changed the drug utilization patterns [41]. Feedback mechanism has been developed for patients and prescribers in MATES. Guidelines for various diseases have also been given to patients as well as prescribers. Furthermore, portals have been established for log-in and in this way, uniform approach of treatment of various health problems have been addressed. Authors recommend the development of such a useful project in Pakistan for safe and effective use of medicines at the level of patients as well as physicians. Moreover, Authors recommend that besides Government, patients, prescribers; other key partners such as, pharmaceutical industry and medicine distributors should also be taken on board to achieve the goal of quality use of medicine.

## Conclusion

Results of the present study conclude that variation in perception of study participants on certain aspects of prescription suggests the development of policy guidelines in connection with prescribing practices to reduce the cost of drugs. The reduction in the cost of drugs can be done by reducing forced prescribing and indirectly reduction in adverse health outcome by decreasing the liaison and price driven un-necessary prescribing. To address the issue of availability of drug in the local market and perception of public and health care providers on cost and effectiveness of medicines of multinational and local companies also needs the development of policy frame work at the level of Federal Government and Drug Regulatory Authority of Pakistan. Fixation of uniform prices of different registered brands of drugs for the same generic to reduce the price driven prescriptions as indicated in variant perceptions also needs to be addressed at the Government level. Policy also needs to be developed to highlight at the public level that registered drugs of all the licensed manufacturers are equally effective as all the brands of a particular molecule are being manufactured in accordance with GMP guidelines of the Drug Regulatory Authority of Pakistan.

## Abbreviations

(EDL): Essential Drug List
(GMP): Good Manufacturing Practice
(MATES): Medicine Advice Therapeutic Education Services
(MS): Medical Stores
(PMDC): Pakistan Medical and Dental Council
(PPC): Pakistan Pharmacy Council
(RMPs: Registered Medical Practitioners
(SPSS): Statistical Package Social Sciences®
(WHO): World Health Organization
